# Effects of Chronic Intermittent Hypoxia and Chronic Sleep Fragmentation on Gut Microbiome, Serum Metabolome, Liver and Adipose Tissue Morphology

**DOI:** 10.3389/fendo.2022.820939

**Published:** 2022-02-01

**Authors:** Fan Wang, Juanjuan Zou, Huajun Xu, Weijun Huang, Xiaoman Zhang, Zhicheng Wei, Xinyi Li, Yupu Liu, Jianyin Zou, Feng Liu, Huaming Zhu, Hongliang Yi, Jian Guan, Shankai Yin

**Affiliations:** ^1^ Department of Otolaryngology-Head and Neck Surgery and Shanghai Key Laboratory of Sleep Disordered Breathing and Otolaryngology Institute of Shanghai Jiao Tong University, Shanghai Jiao Tong University Affiliated Sixth People’s Hospital, Shanghai, China; ^2^ Department of Otorhinolaryngology and National Health Commission (NHC) Key Laboratory of Otorhinolaryngology, Shandong University Affiliated Qilu Hospital, Jinan, China

**Keywords:** metabolome, microbiome, lipid metabolic disorders, chronic intermittent hypoxia, chronic sleep fragmentation

## Abstract

Chronic intermittent hypoxia (CIH) and chronic sleep fragmentation (CSF) are two cardinal pathological features of obstructive sleep apnea (OSA). Dietary obesity is a crucial risk intermediator for OSA and metabolic disorders. Gut microbiota affect hepatic and adipose tissue morphology under conditions of CIH or CSF through downstream metabolites. However, the exact relationship is unclear. Herein, chow and high-fat diet (HFD)-fed mice were subjected to CIH or CSF for 10 weeks each and compared to normoxia (NM) or normal sleep (NS) controls. 16S rRNA amplicon sequencing, untargeted liquid chromatography-tandem mass spectrometry, and histological assessment of liver and adipose tissues were used to investigate the correlations between the microbiome, metabolome, and lipid metabolism under CIH or CSF condition. Our results demonstrated that CIH and CSF regulate the abundance of intestinal microbes (such as *Akkermansia mucinphila*, *Clostridium* spp., *Lactococcus* spp., *and Bifidobacterium* spp.) and functional metabolites, such as tryptophan, free fatty acids, branched amino acids, and bile acids, which influence adipose tissue and hepatic lipid metabolism, and the level of lipid deposition in tissues and peripheral blood. In conclusion, CIH and CSF adversely affect fecal microbiota composition and function, and host metabolism; these findings provide new insight into the independent and synergistic effects of CIH, CSF, and HFD on lipid disorders.

## 1 Introduction

Obstructive sleep apnea (OSA) is a common sleep-disordered breathing disease that affects almost 1 billion adults worldwide (1). OSA is associated with high economic and health burdens due to the increased risk of cardiovascular and metabolic diseases (2). Exploring the mechanisms underlying the effects of OSA may lead to improved diagnosis and treatment of the disease and its comorbidities. OSA is characterized by recurrent episodes of airway collapse during sleep that cause chronic intermittent hypoxia (CIH) and sleep disruption due to recurrent arousals, called chronic sleep fragmentation (CSF) (3). CIH and CSF are associated with a wide range of neural, hormonal, thrombotic, and metabolic alterations that promoted OSA-related complications (4–6).

In our previous systemic review, we summarized the changes in the metabolome and microbiota that may play an integral role as intermediary factors in the pathophysiology of OSA and related cardiovascular, metabolic, and neurological complications (7). In rodent studies, CIH and CSF induce dysbiosis of the gut microbial community and serum metabolomics changes. In particular, CIH-exposed models showed an altered Firmicutes/Bacteroidetes (F/B) ratio, reduced abundance of Proteobacteria and Clostridia, and changes in molecular metabolites (>22%) suggestive of excessive production of reactive oxygen species (ROS) and free fatty acids (FFA) (8–10). In contrast, long-term CSF-induced gut microbiota changes include increased abundances of Lachnospiraceae and decreased abundance of Lactobacillaceae (11). The level of energy metabolism and monoamine hormones were significantly reduced in CSF mice compared to controls (12). However, few previous studies have comprehensively examined the effects of CIH and CSF on gut microbial composition and function, or the association thereof with host metabolism.

In the present study, we explored the effects of CIH and CSF, with or without dietary intervention, on gut microbial composition, metabolic function, and adipose tissue morphology. We aimed to determine how, and to what extent, CIH and CSF affect hepatic and adipose tissue morphology; changes in gut microbiome and serum metabolites associated with CIH and CSF; and the impact of CIH- and CSF-mediated taxonomic and molecular alterations on hepatic and adipose lipid metabolism.

## 2 Materials And Methods

### 2.1 Animals Fed a High-Fat Diet

**Figure 1:**
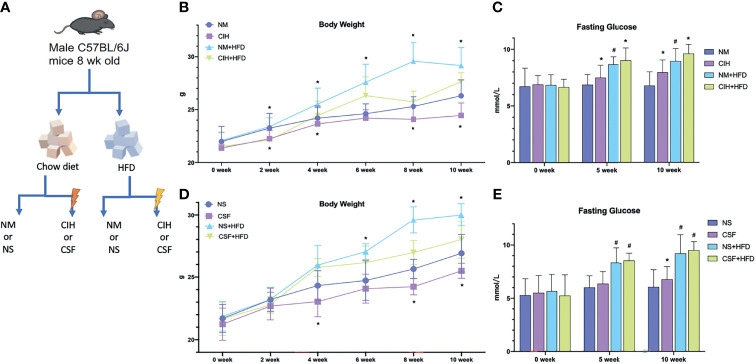
Overall modeling workflow and baseline data. **(A)** CIH and CSF with diet intervention modeling workflow and design. **(B)** Body weights of the chow diet and HFD groups throughout the experiment of CIH exposure. **(C)** The fasting blood glucose levels of the mice in the chow diet and HFD groups during the period of CIH. **(D)** Body weights of the chow diet and HFD groups throughout the experiment of CSF exposure. **(E)** The fasting blood glucose levels of the mice in the chow diet and HFD groups during the period of CSF. Data are expressed as mean ± SEM. Group differences were assessed by using the Mann‐Whitney U test. ^*^P < 0.05 when comparing NM/NS and CIH/CSF groups (NM/NS group vs CIH/CSF group, NM+HFD/NS+HFD group vs CIH+HFD/CSF+HFD group). ^#^P < 0.05 when comparing chow diet and HFD groups (NM/NS group vs NM+HFD/NS+HFD group).

The study protocol was approved by the Institutional Animal Care and Use Committee of Shanghai Jiao Tong University Affiliated Sixth People’s Hospital, China. All experiments were conducted following the Guide for the Care and Use of Laboratory Animals published by the Animal Welfare Committee of the Agricultural Research Organization. Seventy-two male C57BL/6J mice (6 weeks old; Shanghai Laboratory Animal Center, Shanghai, China) were housed in 16 cages (4–5 mice/cage) under standard conditions (temperature: 21 ± 1°C; relative humidity: 50 ± 10%) with a regular 14‐h light: 10‐h dark cycle (lights on at 7:00 am). After adaptation for 2 weeks, mice (8 weeks old) were randomly divided into eight groups: normoxia (NM; n = 8), CIH (n = 10), HFD (n = 8), CIH+HFD (n = 10), normal Sleep (NS; n = 9), CSF (n = 9), NS+HFD (n = 9) and CSF+HFD (n = 9) groups (**Figure 1A**).

The mice had free access to food and water for 10 weeks. The chow diet group was fed a low-calorie diet (10% calories from fat), whereas the HFD group was fed a eutrophic diet with 59% calories derived from coconut oil (13).

### 2.2 CIH Exposure

CIH was induced in identical chambers (Oxycycler model A84; BioSpherix, Redfield, NY, USA) for 8h/day, from 8:00 am to 4:00 pm, for 10 weeks. The O_2_ concentration in the chamber was continuously measured using an O_2_ analyzer. Hypoxia was induced with 10% O2 for approximately 240 s, followed by 21% O2 for 120 s, as previously described (14). For NM controls, 10% O2 (hypoxia) was replaced by 21% O2 (air), while the other conditions were similar to those for the CIH groups.

### 2.3 CSF Intervention

Continuous CSF was induced for 10 weeks by using big large, modified, multiple cages equipped with a fixed platform and twirling pole (MaxQ 2000; ThermoFisher Scientific, Waltham, MA, USA), controlled using a timer programmed for random rotation (H3CR-F8-300; Omron Corp., Kyoto, Japan). CSF was induced by rotating the pole for 10 s after every 110 s at a speed of 110 rpm for the entire day to disturb sleeping mice (15).

### 2.4 Hepatic and Adipose Tissue Morphology

The body weight of all mice was recorded weekly and blood glucose (BG) levels were measured every 5 weeks after fasting for 12 h using a glucose analyzer (Accu-Chek; Roche, Rotkreuz Switzerland) over the tail vein. After the 10-week exposure period and 12-h solid and liquid fast, the mice were sacrificed *via* an overdose of sodium pentobarbital (100 mg/kg, i.p.). Then, samples from the liver, inguinal white adipose tissue (IWAT), perirenal white adipose tissue (PWAT), and scapula brown adipose tissue (BAT) were harvested and weighed. Liver tissues were embedded in Tissue-Tek OCT Compound (Sakura Finetek, Torrance, CA, USA), sliced into cryosections (8-μm-thick) and stained with Oil Red O (Sigma-Aldrich, St. Louis, MO, USA) to assess the fatty droplet content. The stained liver cells were analyzed using Image Pro-Plus 6.0 (Media Cybernetics, Bethesda, MD, USA). Adipose tissues were fixed in 10% formalin, embedded in paraffin wax, sectioned (5 μm), and stained with hematoxylin and eosin. Hematoxylin and eosin staining was used to evaluate the sizes of subcutaneous and visceral white adipose tissue (WAT) and BAT. Mean cell size and size distribution was determined for WAT. Because it was difficult to isolate the BAT cells due to cytoplasmic lipid accumulation, the lipid area of BAT was quantified. The lipid area and cell size were calculated using MetaMorph imaging software (Molecular Devices, Downingtown, PA, USA).

### 2.5 Microbiome Assessment

Sampling time of intestinal contents was on the morning after 10-week intervention and overnight fasting and water restriction. The intestinal contents were collected from the middle section of colon after anesthesia and laparotomy. Fecal samples were collected from all groups for microbiota 16S rRNA analysis. The microflora were detected by Lianchuan Biotechnology Co., Ltd. (Hangzhou, China). Bacterial genomic DNA was obtained from frozen colon contents using the QIAamp DNA Stool Mini Kit (51504; Qiagen, Germantown, MD, USA), according to the manufacturer’s instructions. Successful DNA isolation was confirmed by agarose gel electrophoresis. DNA samples were amplified by polymerase chain reaction (PCR) using bar‐coded primers flanking the V3–V4 region of the 16S rRNA gene. PCR was performed using a thermocycler under the following conditions: 1 pre-denaturation cycle at 94°C for 4 min, 25 cycles of denaturation at 94°C for 30 s, annealing at 50°C for 45 s, elongation at 72°C for 30 s, and 1 post-elongation cycle at 72°C for 5 min. The PCR amplicon products were separated on 0.8% agarose gels and extracted. Only PCR products without primer dimers and contaminant bands were used for sequencing by synthesis. High‐throughput pyrosequencing of the PCR products was performed using the Illumina MiSeq platform (Illumina, Inc., San Diego, CA, USA). Paired‐end reads of the original DNA fragments were excluded from the analysis if they did not well-match a 12‐base Golay barcode (one or no errors), the read overlap was < 35 bases, the overlapped region differed by more than 15%, or there were more than three base calls below Q20. Bacterial operational taxonomic units (OTUs) were created by clustering the reads at 97% identity in the Quantitative Insights into Microbial Ecology (QIIME; http://qiime.org/scripts/pick_otus.html) database.

### 2.6 Metabolome Assessment

Venous blood samples were obtained from mice after overnight fasting. Peripheral blood was allowed to clot for 30 min at room temperature and centrifuged at 3,000 rpm for 10 min to collect the supernatant. Serum samples were immediately stored as aliquots at −80°C prior to further sample preparation and analysis. A total of 50 µL of serum from each of the eight groups was used for metabolic profiling. Untargeted metabolites were detected by Lianchuan Biotechnology. Briefly, serum metabolites were measured by direct-injection electrospray tandem mass spectrometry (MS/MS) using a Micromass Quattro Micro liquid chromatography (LC)-MS system (Waters-Micromass, Milford, MA, USA) equipped with a model HTS-PAL 2777 autosampler (Leap Technologies, Carrboro, NC, USA), model 1525 high-performance liquid chromatography (HPLC) solvent delivery system (Agilent Technologies, Palo Alto, CA, USA), and MassLynx data system (version 4.0; Waters-Micromass). For each batch, periodic analysis of the same pooled quality control (QC) sample was performed to detect variations within and between experiments (16). The **Supplementary Tables S3**, **S4** summarized detected secondary melabolites including the amino acids, organic carbohydrate acids and lipids molecules.

### 2.7 Statistical Analysis

Taxonomy abundance was normalized to the summed OTUs of each sample. Principal coordinate analysis (PCoA) of β-diversity was performed; the (unpaired) Wilcoxon rank-sum test was used to identify the most diverse taxa. Phylogenetic Investigation of Communities by Reconstruction of Unobserved States (PICRUSt) analysis was applied to metagenome functions predicted from the normalized OTUs for Kyoto Encyclopedia of Genes and Genomes (KEGG) orthologs (17). The Mann‐Whitney U test and Spearman’s correlation were performed using SPSS software (version 26.0; IBM Corp., Armonk, NY, USA). Data are expressed as mean ± SEM. Significance was set at p < 0.05^*^ or p < 0.01^**^.

## 3 Results

### 3.1 Effects of CIH and CSF on Body, Liver, and Adipose Tissue Weight, and Glucose Level

All mice survived and were kept in good condition during the 10-week-long CIH or CSF exposure with or without a HFD (**Figure 1A**). The body weight significantly decreased after 2 weeks under CIH in both the chow- and HFD-fed groups compared to the control groups (**Figure 1B**). Similarly, a 4-week-long CSF intervention down-regulated the weight gain in mice irrespective of the diet (**Figure 1D**). The body weight was significantly higher in the NM and NS+HFD groups than the other groups (**Figures 1B, D**). In later stages, the change in body weight in the eight groups decreased. Fasting BG level increased with CIH and HFD interventions, and was higher in the CIH+HFD group compared to the other groups (**Figure 1C**). The fasting BG level was significantly higher in the NS+HFD compared to CSF group (**Figure 1E**).

**Figure 2:**
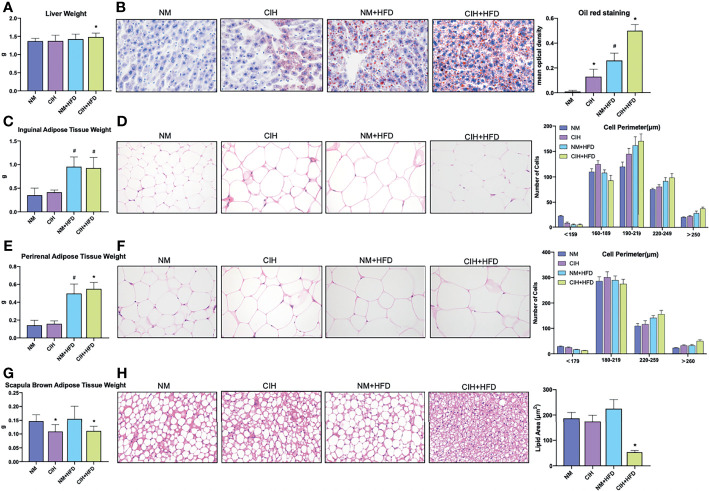
Profiles of liver tissue, abdominal subcutaneous and visceral white adipose tissue, and scapula brown adipose tissue of mice exposed to CIH intervention. **(A)** Liver weights of the mice in the chow diet and HFD groups pre- and post- CIH. **(B)** Oil red staining of liver tissue and mean optical density of the mice in the chow diet and HFD groups pre- and post- CIH. **(C)** Inguinal adipose tissue (Abdominal subcutaneous white adipose tissue) weights of the mice in the normal and HFD groups before and after CIH. **(D)** Hematoxylin-eosin staining and the distribution of adipocyte perimeters of inguinal adipose tissue of the chow diet-fed and HFD-fed mice before and after CIH. **(E)** Perirenal adipose tissue (abdominal visceral white adipose tissue) weights of the mice in the chow diet and HFD groups pre- and post- CIH. **(F)** Hematoxylin-eosin staining and the distribution of adipocyte perimeters of perirenal adipose tissue of the mice and brown fat weight of the chow diet-fed and HFD-fed mice before and after CIH. **(G)** Scapula brown adipose tissue weights of the mice in the chow diet and HFD groups pre- and post- CIH. **(H)** Hematoxylin-eosin staining and lipid area of scapula brown adipose tissue of the mice in the chow diet and HFD groups pre- and post- CIH. Data are expressed as mean ± SEM. Group differences were assessed by using the Mann‐Whitney U test. ^*^P < 0.05 when comparing NM and CIH groups (NM group vs CIH group, NM+HFD group vs CIH+HFD group). ^#^P < 0.05 when comparing chow diet and HFD groups (NM group vs NM+HFD group). Original magnification: 400 ×.

Red oil staining of the liver tissues (**Figure 2B**) revealed that 10-week CIH exposure sharply increased microbubble lipid droplets in liver cells, particularly around the hepatic sinus, compared to the NM group. HFD-fed mice showed marked homogeneous steatosis in parenchymal hepatocytes. The CIH+HFD group demonstrated excessive deposition of microbubble and diffuse bullae lipid droplets, disordered hepatic cords, and higher liver weight compared to the control group (**Figures 2A, B**).

**Figure 3:**
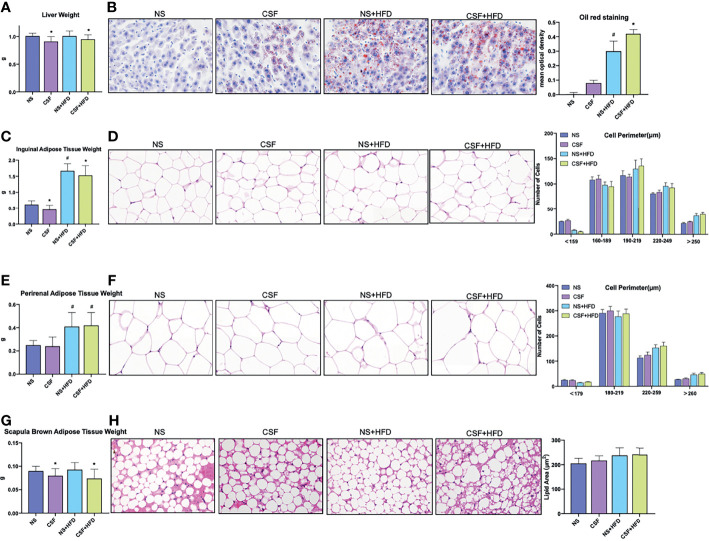
Profiles of liver tissue, abdominal subcutaneous and visceral white adipose tissue, and scapula brown adipose tissue of mice exposed to CSF intervention. **(A)** Liver weights of the mice in the chow diet and HFD groups pre- and post- CSF. **(B)** Oil red staining of liver tissue and mean optical density of the mice in the chow diet and HFD groups pre- and post- CSF. **(C)** Inguinal adipose tissue (Abdominal subcutaneous white adipose tissue) weights of the mice in the normal and HFD groups before and after CSF. **(D)** Hematoxylin-eosin staining and the distribution of adipocyte perimeters of inguinal adipose tissue of the chow diet-fed and HFD-fed mice before and after CSF. **(E)** Perirenal adipose tissue (abdominal visceral white adipose tissue) weights of the mice in the chow diet and HFD groups pre- and post- CSF. **(F)** Hematoxylin-eosin staining and the distribution of adipocyte perimeters of perirenal adipose tissue of the mice and brown fat weight of the chow diet-fed and HFD-fed mice before and after CSF. **(G)** Scapula brown adipose tissue weights of the mice in the chow diet and HFD groups pre- and post- CSF. **(H)** Hematoxylin-eosin staining and lipid area of scapula brown adipose tissue of the mice in the chow diet and HFD groups pre- and post- CSF. Data are expressed as mean ± SEM. Group differences were assessed by using the Mann‐Whitney U test. ^*^P < 0.05 when comparing NS and CSF groups (NS group vs CSF group, NS+HFD group vs CSF+HFD group). ^#^P < 0.05 when comparing chow diet and HFD groups (NS group vs NS+HFD group). Original magnification: 400 ×.

Liver weight (**Figure 3A**) markedly decreased after 10 weeks of CSF with or without HFD. In contrast to the CIH group, the CSF group did not demonstrate increased deposition of microbubble lipid droplets in liver cells (**Figure 3B**). The liver morphology of CSF+HFD mice was similar to that of HFD mice, except for hepatic steatosis. Moreover, tissue distant from the hepatic sinuses showed vacuolar degeneration and sporadic nucleolysis in the CSF+HFD group.

The weights of abdominal subcutaneous and visceral WAT were similar in all chow-fed mice, irrespective of CIH stimulation (**Figure 2C, E**). Hematoxylin and eosin staining showed enlarged lipid droplets in white adipocytes in the CIH mice (**Figure 2D, F**). The WAT cells were vacuolated and disorganized in HFD-fed mice. The PWAT weight was significantly higher in the CIH+HFD compared to HFD mice (**Figure 2E**). The cytoderm of hypertrophic IWAT in the CIH+HFD group was thin and irregularly shaped (**Figure 2D**).

CSF intervention was associated with reduced IWAT weight (**Figure 3C**), but not morphological variation of adipose cells (**Figure 3D**). The PWAT weight appeared to be influenced by the HFD diet, but not by CSF exposure (**Figure 3E**). The majority of WAT cells were transformed from smaller adipose cells to loose cells in the HFD and CSF+HFD groups compared to the controls (**Figures 3D, F**).

Weight of the scapula BAT significantly decreased in the CIH and CSF groups despite HFD (**Figures 2G**, **3G**). Fibrosis and lipid fragmentation were observed in the BAT due to the combined effects of HFD and CIH or CSF stimulation (**Figures 2H**, **3H**).

### 3.2 Effects of CIH and CSF on Fecal Microbiota

#### 3.2.1 Effects of CIH on Fecal Microbiota

**Figure 4:**
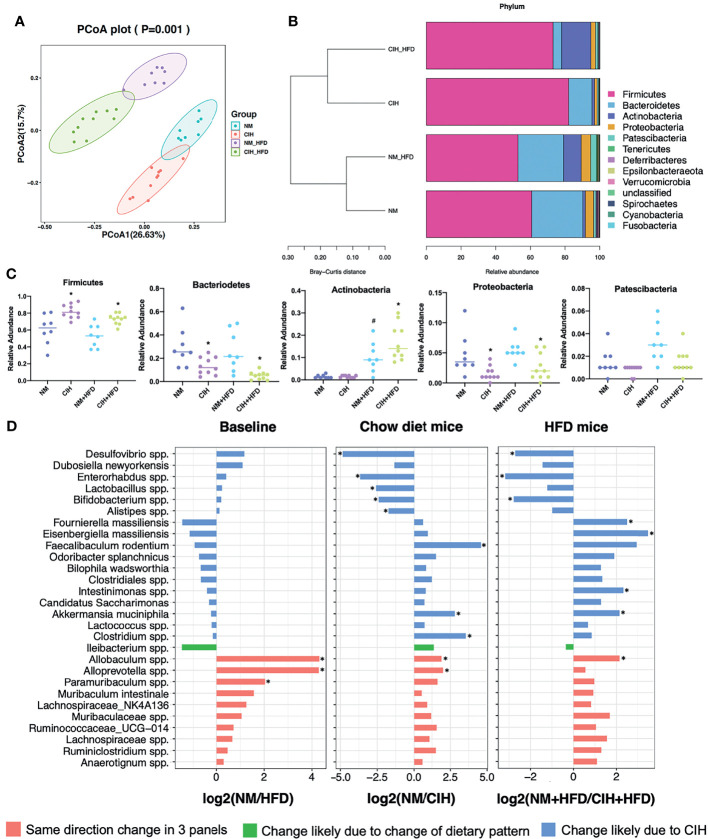
Effects of CIH on fecal microbiota. **(A)** PCoA plot generated by using microbiota OTU metrics based on the Bray‐Curtis similarity for the NM, CIH, NM+HFD, CIH+HFD groups (n = 8-10/group). **(B)** The hierarchical cluster based on the Bray‐Curtis similarity of the samples from the NM, CIH, NM+HFD, CIH+HFD groups. The bar plot shows the abundance of each phylum in each sample. **(C)** The 5 most abundant phyla in the NM, CIH, NM+HFD, CIH+HFD groups. Data are expressed as mean ± SEM. Group differences were assessed by using the Mann‐Whitney U test. ^*^P < 0.05 when comparing NM vs CIH, or NM+HFD vs CIH+HFD, ^#^P < 0.05 when comparing NM vs NM+HFD. **(D)** Fold changes of the annotated microbes at the genus level. The data of log_2_FC are expressed as mean. Group differences were assessed by using the Mann‐Whitney U test. ^*^P < 0.05.

The PCoA plot demonstrated that the NM and CIH groups were separated along the PC1 axis, whereas the NM+HFD and CIH+HFD groups were separated along the PC2 axis (**Figure 4A**). We further evaluated group similarities at the phylum level *via* hierarchical clustering of the four groups (**Figure 4B**). CIH significantly changed the microbial composition in both chow- and HFD-fed mice. As shown in the PCoA diagram (**Figure 4A**), the difference between CIH and NM groups was more significant than that between CIH+HFD and NM+HFD groups. Analysis of phylum abundance revealed that the predominant bacteria in the cecum were Firmicutes, Bacteroidetes, Actinobacteria, Proteobacteria, and Patescibacteria (**Figure 4C**). Moreover, CIH significantly increased the abundance of Firmicutes and Proteobacteria, and decreased that of Bacteroidetes in both chow- and HFD-fed mice. The abundance of Actinobacteria was increased in the NM+HFD and CIH+HFD groups as a result of the change in dietary pattern or additive effect of CIH.

We further evaluated differences in the cecal microbial species at the genus level. Twenty-eight microbes were selected, as there was no “0” relative abundance value in any CIH model. **Figure 4D** illustrates the log_2_ fold change (log_2_FC) in the NM/NM+HFD (baseline difference), NM/CIH (CIH‐induced changes in chow-fed mice), and NM+HFD/CIH+HFD (CIH-induced changes in HFD mice) groups. Red bars indicate the same direction of log_2_FC change in the three NM/CIH and NM+HFD/CIH+HFD pairwise group comparisons. The highest log_2_FC value was observed for *Allobaculum* spp. Green bars indicate the same direction of log_2_FC change for the NM/NM+HFD and NM+HFD/CIH+HFD groups; the changes in *Ileibacterium* spp. were primarily induced by dietary patterns. Blue bars indicate microbes with the same direction of log_2_FC change for the NM/CIH and NM+HFD/CIH+HFD groups, suggesting that the variation may have been induced by CIH. Importantly, CIH significantly increased the abundances of *Desulfovibrio* spp., *Enterohabdus* spp., and *Bifidobacterium* spp. in chow- and HFD-fed mice (log_2_FC < 0; p < 0.05). CIH alone significantly increased the abundances of *Lactobacillus* spp. *and Alistipes* spp., while decreasing the abundances of *Feacalibaculum rodentium* and *Clostridium* spp. (log_2_FC > 0; p < 0.05). The CIH+HFD intervention down-regulated the abundances of *Fournierella massiliensis*, *Eisenbergiella massiliensis*, *Intestinimonas* spp., and *A. muciniphila.*

#### 3.2.2 Effects of CSF on Fecal Microbiota

**Figure 5:**
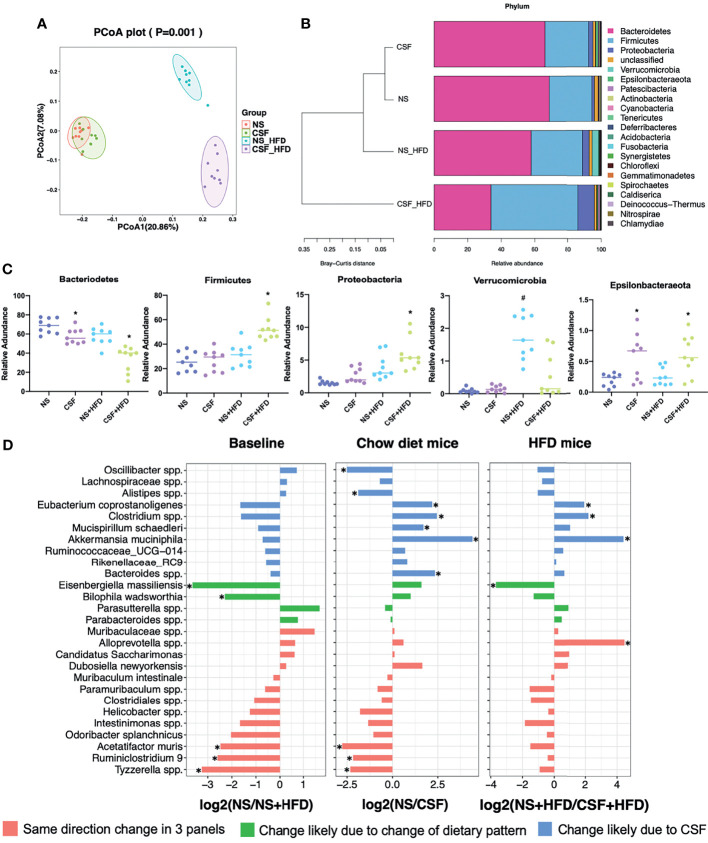
Effects of CSF on fecal microbiota. **(A)** PCoA plot generated by using microbiota OTU metrics based on the Bray‐Curtis similarity for the NS, CSF, NS+HFD, CSF+HFD groups (n = 9/group). **(B)** The hierarchical cluster based on the Bray‐Curtis similarity of the samples from the NS, CSF, NS+HFD, CSF+HFD groups. The bar plot shows the abundance of each phylum in each sample. **(C)** The 5 most abundant phyla in the NS, CSF, NS+HFD, CSF+HFD groups. Data are expressed as mean ± SEM. Group differences were assessed by using the Mann‐Whitney U test. ^*^P < 0.05 when comparing NS vs CSF, or NS+HFD vs CSF+HFD, ^#^P < 0.05 when comparing NS vs NS+HFD. **(D)** Fold changes of the annotated microbes at the genus level. The data of log_2_FC are expressed as mean. Group differences were assessed by using the Mann‐Whitney U test. ^*^P < 0.05.

The PCoA plot (**Figure 5A**) showed that the NS and CSF groups were partially separated along the PC1 axis, whereas the NS+HFD and CSF+HFD groups were fully separated along the PC2 axis. “Stacked clustering” (**Figure 5B**) of the four groups revealed that CSF exposure slightly altered the microbial composition of chow-fed mice compared to HFD-fed mice. In contrast, the microbial composition was significantly changed after the combined CSF+HFD intervention.

Analysis of the phyla of microbiota demonstrated that Bacteroidetes, Firmicutes, Proteobacteria, Verrucomicrobia, and Epsilonbacteraeota dominated the fecal bacterial population (**Figure 5C**). CSF exposure significantly decreased the abundance of Bacteroidetes, but increased the abundances of Firmicutes and Proteobacteria in HFD-fed mice. Irrespective of the dietary pattern, the abundance of Epsilonbacteraeota increased after CSF.

Twenty-seven species labeled at the genus level were selected to compare the log_2_FC (**Figure 5D**) between groups. CSF significantly reduced the abundances of *Eubacterium coprostanoligenes*, *Clostridium* spp., and *A. muciniphila*, irrespective of dietary intervention. CSF exposure alone significantly upregulated *Oscillibacter* spp. and *Alistipes* spp., and downregulated *Mucispirillum schaedleri* and *Bacteroides* spp.

### 3.3 Effects of CIH and CSF on Serum Metabolome

#### 3.3.1 Effect of CIH on Serum Metabolome

**Figure 6:**
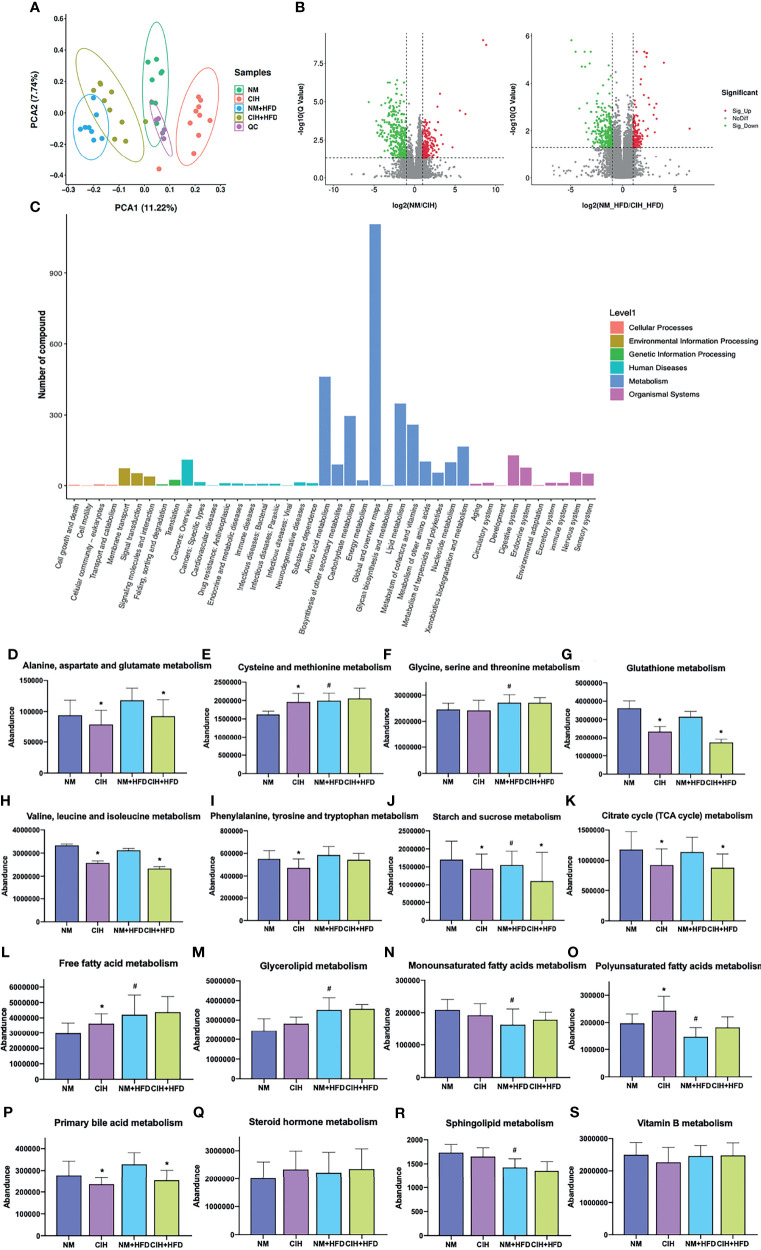
Effects of CIH on the serum metabolome. **(A)** The PCA plot of metabolites generated by using the metabolite intensity from the NM (n = 8), CIH (n = 10), NM+HFD (n = 8), and CIH+HFD (n = 10) groups. **(B)** Volcano plots of metabolites with FDR < 0.05 and fold changes of > 4/3 or < 3/4 when comparing NM vs CIH, or comparing NM+HFD vs CIH+HFD. **(C)** Enrichment of activated pathways induced by alterations of metabolites in NM, CIH, NM+HFD and CIH+HFD groups. **(D–S)** The abundance of metabolite pathways from the NM, CIH, NM+HFD and CIH+HFD groups. Data are expressed as mean ± SEM. Group differences were assessed by using the Mann‐Whitney U test. ^*^P < 0.05 when comparing NM vs CIH, or NM+HFD vs CIH+HFD, ^#^P < 0.05 when comparing NM vs NM+HFD.

We detected 16,493 peaks (**Table S1**) in the positive and negative ionization modes, likely induced by CIH. After excluding the natural isotopic peaks, 320 and 516 metabolites (**Supplementary Table S3**) were identified in the positive and negative modes, respectively, including amino acids, carbohydrates, lipid molecules, bile acids (BA), fatty acids and vitamins. Unsupervised principal component analysis (PCA) analysis was used to evaluate the intrinsic metabolic variations. Metabolites from the CIH group were more separated than those from the NM control group. However, metabolites from the CIH+HFD group showed less obvious separation than those from the NM+HFD group, indicating more significant metabolic differences between the CIH and NM groups (**Figure 6A**). Tight clustering of the QC samples indicated good reproducibility across samples. Volcano plots (**Figure 6B**) were constructed to evaluate the differential metabolites between the NM and CIH groups, and between the NM+HFD and CIH+HFD groups. The statistical significance level in the clustering analysis was a false discovery rate < 0.05 (> 4/3- or < 3/4-fold change).

**Figure 6C** shows the enrichment of activated pathways, focusing on metabolism-related pathways. We further analyzed the effects of CIH on host metabolic alterations in terms of the abundance of specific metabolic pathways. The detailed calculation was based on identified secondary metabolites (**Supplementary Tables S3**, including corresponding names of secondary metabolites and attributable KEGG pathways). According to metabolic pathways of amino acids, carbohydrates and lipids, data of the relative abundance of detected secondary metabolites of all mice in each group was extracted. Then the relative abundance of metabolites in some pathways we were interested in was roughly estimated. **Figures 6D–I** shows that CIH inhibited the metabolism of alanine, aspartate, glutamate, glutathione, valine, leucine, and isoleucine (i.e., branched-chain amino acids, BCAAs), despite HFD. CIH or HFD intervention alone promoted cysteine and methionine metabolism. Phenylalanine, tyrosine, and tryptophan metabolism were downregulated by CIH, which was partially reversed by HFD. **Figures 6J, K** shows that CIH inhibited starch and sucrose metabolism, and the citrate cycle. Additionally, **Figures 6L–R** shows that both CIH and HFD increased FFA metabolism. The increased metabolism of glycerolipid, monounsaturated fatty acids (MUFAs), and sphingolipids due to CIH was further increased after HFD. CIH significantly increased polyunsaturated fatty acid (PUFA) metabolism but decreased the level of primary BA, opposite to the effects of HFD. **Figure 6S** shows that therewere no significant differences in the relative abundance of Vitamin B metabolismbefore and after the CIH intervention.

#### 3.3.2 Effect of CSF on Serum Metabolome

**Figure 7:**
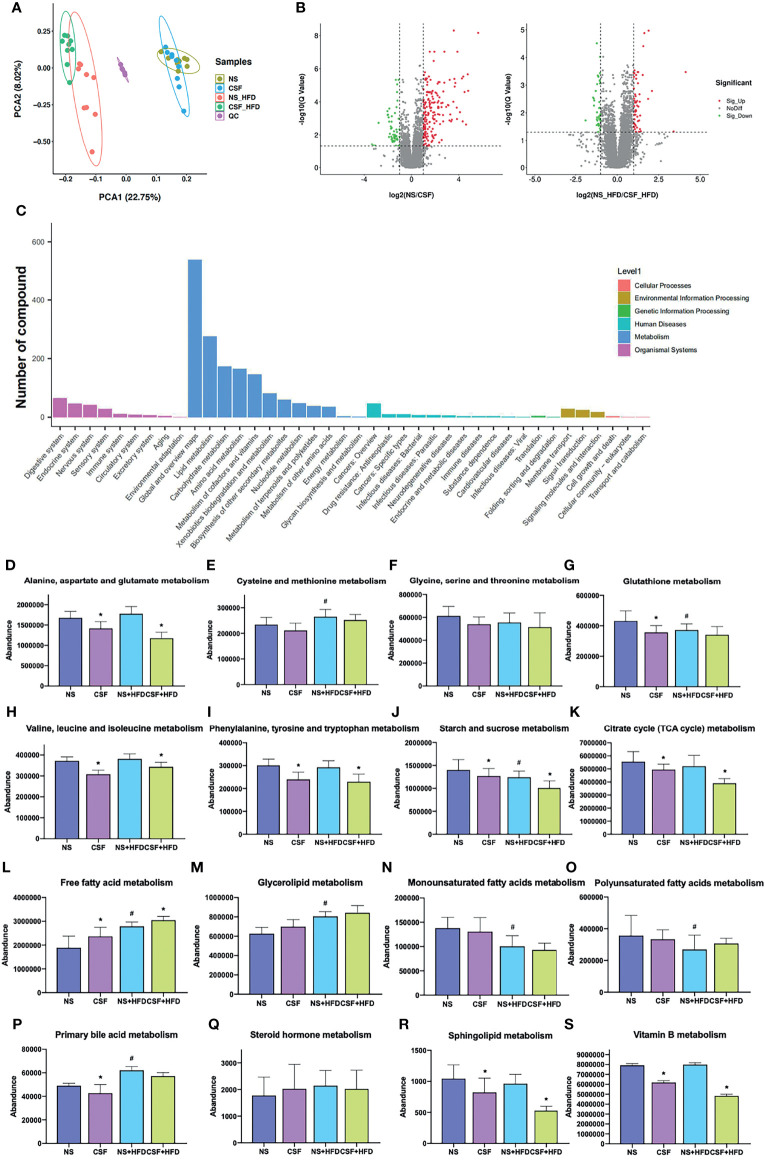
Effects of CSF on the serum metabolome. **(A)** The PCA plot of metabolites generated by using the metabolite intensity from the NS (n = 9), CSF (n = 9), NS+HFD (n = 9), and CSF+HFD (n = 9) groups. **(B)** Volcano plots of metabolites with FDR < 0.05 and fold changes of > 4/3 or < 3/4 when comparing NS vs CSF, or comparing NS+HFD vs CSF+HFD. **(C)** Enrichment of activated pathways induced by alterations of metabolites in NS, CSF, NS+HFD and CSF+HFD groups. **(D–S)** The abundance of metabolite pathways from the NS, CSF, NS+HFD and CSF+HFD groups. Data are expressed as mean ± SEM. Group differences were assessed by using the Mann‐Whitney U test. ^*^P < 0.05 when comparing NS vs CSF, or NS+HFD vs CSF+HFD, ^#^P < 0.05 when comparing NS vs NS+HFD.

In the CSF group, we identified 7,670 natural isotopic peaks (**Supplementary Table S2**), including 280 and 405 metabolites in positive and negative ionization modes (**Table S4**), respectively. The PCA plot (**Figure 7A**) showed that metabolites from the CSF+HFD group were more separated than those from the NS+HFD group, whereas those from the CSF group showed less separation than those from the NS control group, suggesting fewer metabolic differences between the latter two groups.

We explored the impact of CSF on host serum metabolic alterations using Volcano plots (**Figure 7B**) and enrichment of KEGG metabolic pathways (**Figure 7C**). The rough estimation of relative abundance of metabolites in some pathways we focused on in **Figure 7** was based on identified secondary metabolites (**Supplementary Tables S4**, including names of secondary metabolites and information of corresponding KEGG pathways). **Figures 7D–I** showed that CSF inhibited the metabolism of alanine, aspartate, glutamate, glutathione, BCAAs, phenylalanine, tyrosine, and tryptophan. CSF had a synergistic effect with HFD, suppressing starch and sucrose metabolism, and the citrate cycle (**Figures 7J, K**). Additionally, CSF significantly increased the level of FFAs and decreased the levels of primary BA and sphingolipids (**Figures 7L–R**). **Figure 7S** shows that vitamin B metabolism was inhibited by CSF.

### 3.4 Effects of CIH and CSF on Microbial Function

**Figure 8:**
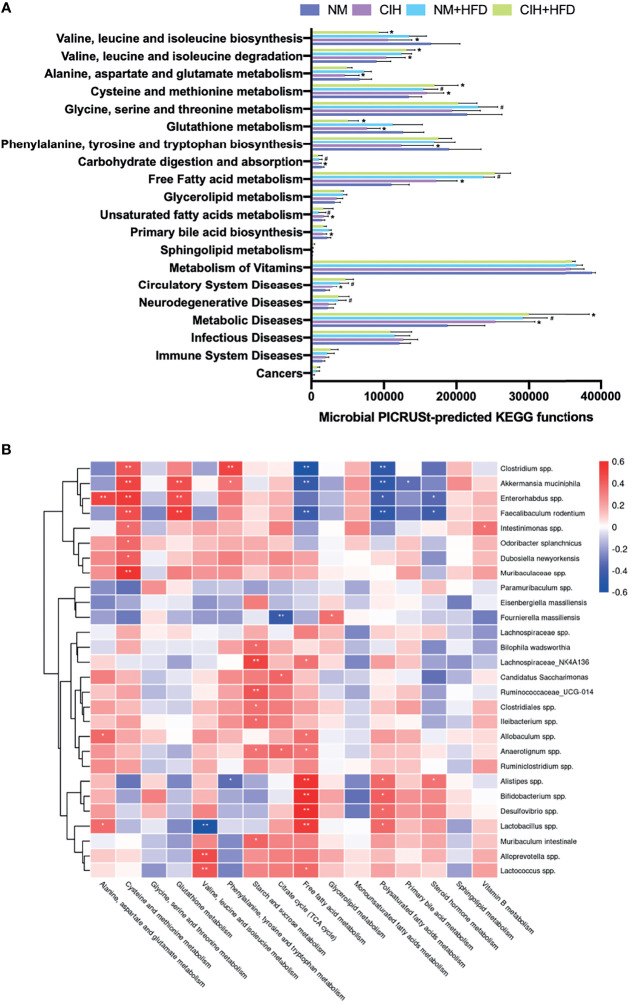
CIH-related fecal microbiota functional changes and correlations between the host metabolome and fecal microbiome. **(A)** Microbial PICRUSt‐predicted KEGG functions relevant to metabolism and diseases. Data are expressed as mean ± SEM. Group differences were assessed by using the Mann‐Whitney U test. ^*^P < 0.05 when comparing NM vs CIH, or NM+HFD vs CIH+HFD. ^#^P < 0.05 when comparing NM vs NM+HFD. **(B)** Spearman correlations of the relative abundance of fecal microbial genus or species and the abundance of metabolite pathways in host serum (n = 36). The r values are represented by gradient colors, with red cells indicating positive correlations and blue cells indicating negative correlations. ^*^P < 0.05, and ^**^P < 0.01.

**Figure 9:**
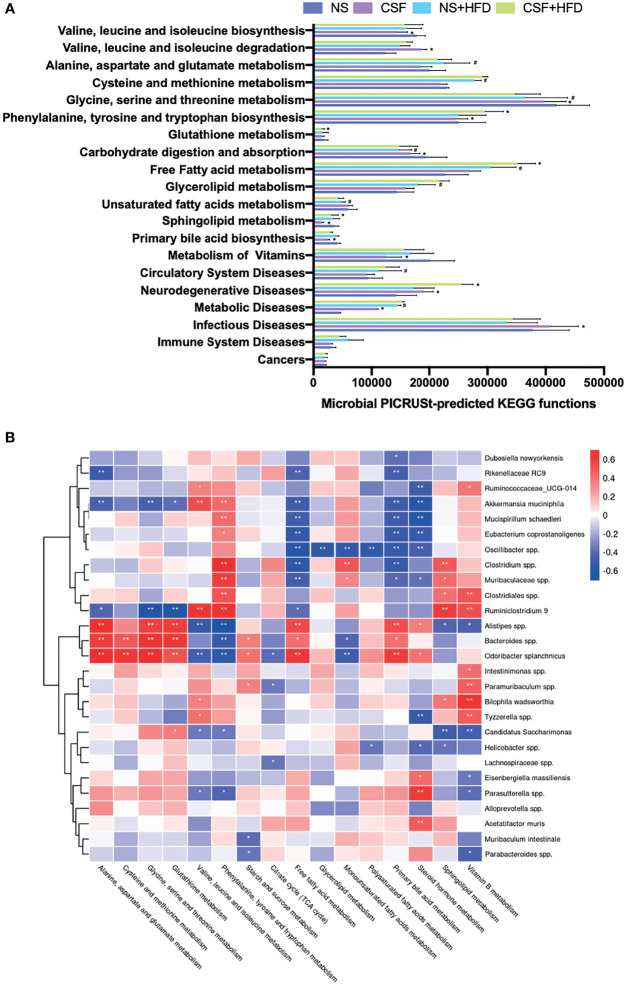
CSF-related fecal microbiota functional changes and correlations between the host metabolome and fecal microbiome. **(A)** Microbial PICRUSt‐predicted KEGG functions relevant to metabolism and diseases. Data are expressed as mean ± SEM. Group differences were assessed by using the Mann‐Whitney U test. ^*^P < 0.05 when comparing NS vs CSF, or NS+HFD vs CSF+HFD. ^#^P < 0.05 when comparing NM vs NM+HFD. **(B)** Spearman correlations of the relative abundance of fecal microbial genus or species and the abundance of metabolite pathways in host serum (n = 36). The r values are represented by gradient colors, with red cells indicating positive correlations and blue cells indicating negative correlations. ^*^P < 0.05, and ^**^P < 0.01.

We evaluated the function of gut microbiota using KEGG and PICRUSt analyses. Group differences in microbial function related to the metabolism of amino acids, carbohydrates, lipids, BAs, and FFAs. **Figure 8A** shows that CIH significantly inhibited BCAA biosynthesis but promoted their degradation, similar to the effects of CSF (**Figure 9A**). Similarly, CIH or CSF exposure significantly inhibited alanine, aspartate, glutamate, phenylalanine, tyrosine, tryptophan, and glutathione metabolism, which was attenuated by HFD. Both the CIH and HFD interventions significantly increased the metabolism of cysteine, methionine, and FFAs, whereas they inhibited glutathione metabolism and carbohydrate digestion and absorption. CIH significantly increased the levels of MUFAs and decreased the level of primary BA, opposite to the effects of HFD. CIH in conjunction with HFD predisposed the mice to metabolic and cardiovascular diseases (CVDs). Similarly, CSF reduced the metabolism of glycine, serine, threonine, and primary BAs, and inhibited carbohydrate digestion and absorption. In particular, CSF exposure substantially inhibited the metabolism of sphingolipids and vitamin B, thereby predisposing the mice to neurodegenerative diseases (NDDs) and infectious diseases (IDs).

### 3.5 Correlations of Microbiota Composition With Host Metabolism and Hepatic and Adipose Tissue Phenotypes

**Figure 10:**
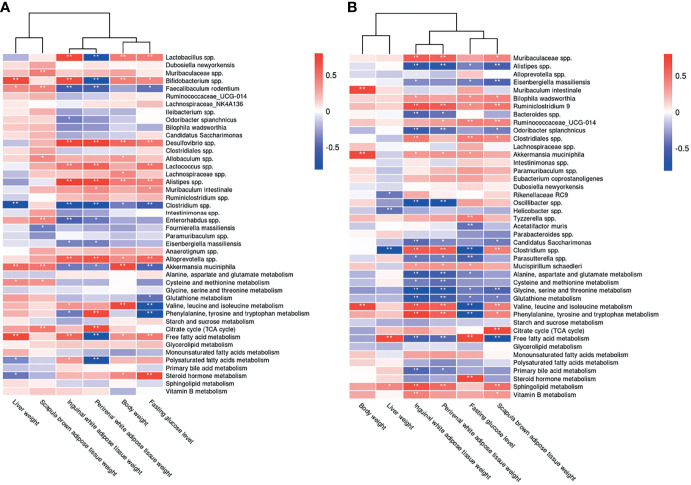
Correlations between the host metabolome, fecal microbiome and hepatic- and adipose- phenotype. **(A)** Spearman correlations of the relative abundance of fecal microbial genus or species, the abundance of metabolite pathways and phenotype of obesity and fasting glucose in host serum of 4 CIH-related modeling groups (n = 36). **(B)** Spearman correlations of the relative abundance of fecal microbial genus or species, the abundance of metabolite pathways and phenotype of obesity and fasting glucose in host serum of 4 PSD-related modeling groups (n = 36). The r values are represented by gradient colors, with red cells indicating positive correlations and blue cells indicating negative correlations. ^*^P < 0.05, and ^**^P < 0.01.

We evaluated the relative abundance of fecal microbes and metabolic pathway activation in CIH host serum and hepatic and adipose tissue phenotypes (**Figures 8B**, **10A**). Several microbes, such as *A. muciniphila*, *Bifidobacterium* spp., *Lactobacillus* spp., and *Clostridium* spp., were significantly correlated with multiple host metabolic pathways, such as the TCA cycle and metabolism of starch, sucrose, FFA, glutathione, and primary BAs.

CSF stimulation was associated with changes in the abundances of *A. muciniphila*, *M. schaedleri*, *Oscillibacter* spp., *Bacteroides* spp., and *Clostridium* spp., and host metabolic pathways (e.g., BCAAs, BA, and FFA) (**Figures 9B**, **10B**).

The microbiota composition and host metabolism were affected by CIH- and CSF-induced changes in body weight, fasting BG levels, and the weights of WAT, BAT, and liver. These results suggested that the structure of various fecal microorganisms was influenced by CIH and CSF, which was closely related to host metabolism and hepatic and adipose morphology.

## 4 Discussion

In this study, we analyzed OSA-associated pathological changes in intestinal bacteria and metabolites, as well as their correlations with hepatic and adipose tissue morphology and lipid metabolism disorders. The two pathological factors of OSA combined with HFD feeding intervention were used to create the corresponding rodent models. The influence of the above pathological intervention on the composition, abundance, and KEGG-based PICRUSt metagenome functions analyses of intestinal flora was identified by 16S rRNA detection. We also extracted the differential metabolites in serum related to the above pathway through untargeted metabolomics detection data to estimate the impact on the metabolic levels of amino acids, carbohydrates and lipids. Then we conducted correlation analysis on the differential data of the two kinds of omics to see the influence of OSA-related main pathological factors on intestinal bacteria and related metabolites. In the modeling process, we found that CIH, CSF intervention factors obviously caused significant weight loss in mice at the early modeling stage, so we also paid attention to the effects of differential microflora, differential metabolites and lipid deposition in adipose tissue and liver tissue. Therefore, we found that CIH and CSF, with or without HFD-induced dysbiosis of intestinal bacteria and metabolites thereof, may independently or synergistically affect the risk of lipid-related metabolic complications.

Adipose tissue and hepatic lipid metabolism involves a dynamic equilibrium between lipogenesis and lipolysis that maintains lipid homeostasis (18). C57BL/6 mice were the optimal model animal for simulating human lipid metabolism disorders (19). Abnormal lipid metabolism is suggested by body weight changes, as well as changes in the weight of subcutaneous or visceral WAT, visceral BAT and liver tissues, and an abnormal distribution of lipid droplets.

We found that CIH and CSF were associated with weight loss in murine models at the early stage of the experiment, even after HFD supplementation (**Figures 1B, D**), which may correlate with a CIH- and CSF-induced reduction in the abundance of *A. muciniphila* (**Figures 4D**, 5D, 10A and 10B), and a CSF-induced reduction in the abundance of *M. schaedleri* (**Figures 5D**, **10B**). Reduced abundances of the abovementioned bacteria led to decreased density of enteric goblet cells, increased intestinal permeability, impaired digestive function (20) and resulted in weight loss (**Figures 10A, B**). The damaged intestinal barrier allows intestinal microbiome-derived components and metabolites to reach target organs, such as adipose and liver tissues.

Besides CIH- or CSF-related functional changes in intestinal bacteria promoted inhibition of the citrate cycle (**Figures 6K**, **7K**, **8B**, **9B**), a significant quantity of BAT was consumed to meet the energy requirement (**Figures 10A, B**) (21). Additionally, BAT cells contained multiple lipid droplets and abundant mitochondria. Upregulation of mitochondrial uncoupling protein 1 (UCP1) activated BAT-mediated decomposition of triglycerides (TGs) into FFAs (22), producing energy for non-shivering heat generation through β-oxidation (23). CIH and CSF upregulated Firmicutes (**Figures 4C** and **5C**), and CIH significantly upregulated *Bifidobacterium* spp. and *Lactobacillus* spp. (**Figures 4D**, **10A**), which may act as strong peroxisome proliferator-activated receptor-γ (PPAR-γ) activators (24). PPAR-γ activation may upregulate UCP1 expression to promote BAT consumption (25), whereas increase BCAA degradation (Figs. 6H, 7H, 8A and 9A), which in turn affects body mass (**Figures 10A, B**) (26). Similarly, weight loss decreased during the posterior experiments (**Figures 1B, D**). Moreover, the CIH+HFD group had an increased abundance of Actinobacteria (**Figure 4C**), which can synthesize PUFAs (27) from HFD and fatty acid derivatives (**Figure 8A**), thereby activating PPAR-γ (28) to improve BAT metabolism in obese mice (**Figure 10A**).

However, CIH and CSF intervention reduced the abundance of *Clostridium* spp. (**Figures 4D**, **8B**, **5D**, **9B**), leading to the inhibition of tryptophan absorption (**Figures 6I**, **7I**, **8A**, **9A**). *Clostridium* spp. converts raw tryptophan into various indole metabolites, such as 5-hydroxytryptamine (5-HT) and melatonin (29). The indole derivatives not only activate PPAR-γ to promote fatty acid thermogenic energy metabolism in rodent models (30), but also serve as an agonist to the aryl hydrocarbon receptor (AHR) to improve intestinal barrier function (31). For instance, melatonin may normalize the F/B ratio and abundance of *A. mucinphila* (30) to improve microbiome dysbiosis, in addition to regulating the circadian rhythm. Decreased tryptophan-derived indoles due to CIH- or CSF-related intestinal dysbiosis mediate intestinal permeability and BAT activation (**Figures 10A, B**).

Excessive energy intake in our HFD group increased the body weight and caused obesity. HFD and CIH had a significant effect on BAT metabolism. Hypoxia was seen in obese mice (32); after CIH, excessive oxygen consumption during the β-oxidation process was blocked and FFAs were decomposed by BAT (33). We observed changes in the levels of FFAs under CIH and CSF conditions, which were accentuated by HFD supplementation (**Figures 6L**, **7L**). Furthermore, *Bifidobacterium* spp. and *Lactobacillus* spp. (**Figures 8A, B**) were upregulated by CIH. *Clostridium* spp. (**Figures 8A, B**, **9A, B**) was downregulated by CIH and CSF (with or without HFD), resulting in excessive accumulation of FFAs due to altered dietary lipid absorption.

FFAs act as ligands to activate the Toll-like receptor (TLR) 2 pathway (34). FFAs may promote lipid metabolic instability and a pro-inflammatory state. Moreover, CIH, CSF, and HFD increased the levels of the pro-inflammatory bacterial compound lipopolysaccharide (LPS, derived from Gram-negative bacterial membranes), which serves as a ligand to activate TLR4 (35). The TLR family of pattern recognition receptors are associated with innate immunity, inflammation, and apoptosis of adipose tissue (35, 36). Activated TLRs inhibited β-oxidation and significantly increased the expression of pro-inflammatory factors such as monocyte chemoattractant protein-1 (MCP-1), interleukin-6 (IL-6), and tumor necrosis factor-alpha (TNF-α) (37), and the pro-apoptosis protein Bax (38). These autophagy-promoting signals inactivate the mitochondria and lipophagy in BAT (33), consistent with FFA-induced effects on autophagy (**Figures 10A, B**) such as adipose fibrosis and fragmentation of lipid droplets in BAT due to CIH, CSF, and/or HFD (**Figures 2H**, **3H**). BAT inactivation (37) is eventually transformed into a crucial inducer of damage to lipid homeostasis.

In contrast to BAT, WAT cells contain only one lipid droplet and few mitochondria. Importantly, we did not observe WAT proliferation in response to CIH or CSF exposure alone. However, CIH or CSF combined with HFD led to an apparent increase in lipid deposition in IWAT and PWAT. CIH and CSF increased F/B ratio (**Figures 4C** and **5C**), consistent with the upregulation of glycerolipid metabolism (**Figures 6M**, **7M**), which promoted the increased TG deposition seen in WAT (39). Importantly, CSF upregulated the probiotic Oscillibacter spp. (**Figure 5D**), which improves lipid metabolism (including of FFAs and glycerolipids) (**Figure 9B**) and inhibits pathological lipid deposition in IWAT and PWAT (**Figure 10B**) (40). Additionally, a large number of WAT cells were transformed into large vacuolated adipose cells (**Figures 2D, F**, **3F**). Furthermore, previous comparative studies of the expression profiles (41) demonstrated that small-sized adipocytes showed high expression levels of anti-inflammatory factors, such as adiponectin and resistin; conversely, large adipocytes that are insensitive to insulin highly expressed inflammatory factors, such as TNF-α and IL-6. In other words, CIH and CSF+HFD increased the number of pro-inflammatory WAT cells, which may contribute to subsequent disturbance of glucose (**Figures 1C, E**). Therefore, apoptosis signal-regulating kinase1 may be specifically activated by TNF-α (42) or enterobacterial disorders (43). The CIH+HFD group demonstrated abnormal morphology and thin cell walls in the IWAT due to the pro-inflammatory and pro-apoptotic conditions (**Figure 2D**).

Additionally, CIH intervention alone (**Figures 4C, D**) significantly inhibited the probiotics Bacteroidetes and *Faecalibacterium prausnitzii*, and upregulated Actinobacteria and Desulfovibrio, leading to pro-inflammatory intestinal dysbiosis (44). After HFD supplementation, CSF decreased the abundance of Bacteroidetes and increased that of Proteobacteria (**Figure 5C**), leading to pro-inflammatory intestinal microbiota. Combined with increased intestinal permeability, LPS promoted the secretion of MCP-1 from large adipocytes, resulting in macrophage accumulation and conversion into pro-inflammatory M1-type adipocyte macrophages (ATM) in WAT (45). The aggregation of M1-type ATM promoted the inflammatory cascade and C-reactive protein synthesis in the liver (46, 47). Therefore, WAT promoted inflammation, IR, and hepatic lipid deposition on exposure to OSA-related factors.

CIH and CSF-derived excessive ROS production caused oxidative damage to cellular macromolecules and altered the physical and chemical properties of liver membranes. Repeated oxidative stress depleted reduced glutathione (48), an essential free radical scavenger. CIH upregulated *Enterorhabdus* spp. and CSF down-regulated *Bacteroides* spp. (**Figures 4D**, **5D**, **8B**, **9B**), which inhibited glutamate absorption (with no significant change in glycine metabolism) and reduced glutathione synthesis (**Figures 6D**, **7D**, **6F**, **7F**, **8A**, **9A**). These changes significantly reduced the capacity for free radical elimination (49), leading to lysosome and mitochondria damage. HFD supplementation worsened the imbalance in the redox state, thereby leading to lipid peroxidation and subsequent liver steatosis.

Oil red staining demonstrated that CIH and CSF induced micro-vesicular lipid droplet deposition in hepatocytes, whereas HFD led to diffuse macro-vesicular hepatic steatosis (**Figures 2B**, **3B**), suggesting that OSA-related changes and dietary intervention had different and independent impacts on steatosis. CIH remarkably increased the F/B ratio (**Figure 4C**) (8), while CSF significantly inhibited polysaccharide fermentation (**Figures 6J**, **7J**, **8A**, **9A**) by decreasing Bacteroides (**Figures 5D**, **9B**), which decreases short-chain fatty acids (SCFAs) synthesis (50). SCFAs protect the liver by upregulating the AMP protein kinase signaling pathway to activate PPAR-α in hepatic adipose tissue (51). Inhibition of the protective PPAR-α in hepatocytes due to insufficient SCFA synthesis, caused by CIH- or CSF-induced changes in intestinal bacteria and derived metabolites, is the root cause of liver lipid metabolism dyshomeostasis (**Figures 10A, B**) (52).

Both CIH and CSF activated the sympathetic adrenal medulla system to release catecholamines, thereby mobilizing glucose and FFAs (53). However, HFD-induced obesity inhibited sympathetic excitability and induced the formation of lipid droplets in hepaticytes (54). CIH-, CSF-, and HFD-mediated intestinal bacteria dysbiosis resulted in stockpiling FFAs (**Figures 6L**, **7L**, **8A, B**, **9A, B**), thereby exceeding the lipid acidification capacity of mitochondria. This leads to the deposition of FFAs in the liver through the portal vein (55). CIH stimulation promoted the expression of hypoxia-inducible factors-1α (56), and HFD upregulated the levels of stearyl CoA desaturase-1, fatty acid synthase (57), and genes involved in adipogenesis and lipid droplet deposition. Therefore, CIH, CSF, and HFD promoted the use of FFAs as raw materials to synthesize TGs and cholesterol (**Figures 10A, B**), leading to hepatocyte steatosis (**Figures 2B**, **3B**) in non-alcoholic fatty liver disease (57, 58).

Even under physiological conditions, the liver consumes a large quantity of oxygen to convert excess dietary carbohydrates into TGs (58). The dual blood supply of the liver leads to a low physiological oxygen partial pressure, which makes hepatocytes, especially those around the central lobule, extremely sensitive to hypoxia. The morphological examination conducted in this study demonstrated that the hepatic vascular sinus region was the first region affected by CIH (and the most severely affected) (**Figure 2B**).

The liver receives most of its blood supply directly from the intestine, which carries the intestinal flora through the portal vein to the liver. Therefore, the host intestinal microbiome and liver are intrinsically linked (59), such that dysbiosis of the intestinal bacterial and derived metabolites due to CIH and CSF leads to hepatic dysfunction (60). CIH, especially CIH+HFD, significantly upregulated *Bifidobacterium* spp. (**Figure 4D**), which produces endogenous ethanol *via* fermentation to stimulate the oxidative stress in hepatic cells and exacerbates liver inflammation (61). Moreover, bacterial ethanol activated the TLRs (35) in hepatocytes to promote chronic hepatic inflammation due to increased intestinal permeability, and also raised the LPS level (62), consistent with the hepatic morphological changes seen in the CIH+HFD group (i.e., diffuse hepatic steatosis, cord disorders, and fibrosis) (**Figures 2A, B**). CIH- and CSF-related bacterial dysbiosis upregulated LPS in the liver and affected peripheral lipid metabolism (63). Even low-dose LPS increased the synthesis of very-low-density lipoprotein in the liver, and high-dose LPS reduced lipoprotein decomposition and promoted dyslipidemia.

CIH and CSF were associated with increased intestinal permeability and levels of intestinal bacterial TLR ligands, which caused adipose and hepatic tissue inflammation and dyslipidosis (64). In OSA, adipose tissue may be the initial site of metabolic inflammation. Massive release of pro-inflammatory cytokines and activation of M1-type ATMs are the first metabolic derangements seen in OSA, followed by the gradual development of metabolic liver inflammation (65, 66). Considering the complex interactions in the CIH- and CSF-related intestinal microbiota-metabolome-adipose/liver axis, it is hypothesized that once hepatic metabolic inflammation is induced, the action of the liver enhances lipid metabolism disorders.

Dyshomeostasis of lipid and glucose metabolism often occur together. CIH and CSF activated the hypothalamic-pituitary-adrenal (HPA) cortex (67), leading to the release of catecholamines, glucocorticoids, and cortisol (**Figures 6Q**, **7Q**) and an increase in the level of fasting BG (**Figures 1C, E**, **10A, B**). Both CIH and CSF inhibited tryptophan absorption and indoles synthesis, which act as AHR agonists to promote secretion of the intestinal hormone GLP-1 and maintain glucose homeostasis (**Figures 10A, B**) (31). Dysbiosis of the intestinal bacteria due to CIH or CSF alone downregulated the production of primary BAs (**Figures 6P**, **7P**, **8A**, **9A**). The abundances of Bacteroidetes and *Clostridium* spp., which reduced the production of secondary BAs through the 7α-decarboxylation reaction process (68), was down-regulated by CIH and CSF (**Figures 4C** **, D**, **5C, D**). Secondary BAs act through Takeda G protein-coupled receptor 5 and promote GLP-1 release from the intestinal L cells (69). Therefore, insufficient secretion of endogenous GLP-1 due to CIH- and CSF-related changes in the intestinal bacteria and derived metabolites could disrupt glucose homeostasis. Additionally, decreased synthesis and increased consumption of BCAAs derived from the intestinal bacteria in CIH and CSF mice (**Figures 6H**, **7H**, **8A**, **9A**, **10A, B**) positively correlated with IR (70). The CIH-, CSF-, and HFD-related gut microbiota dysbiosis resulted in very high FFAs levels (**Figures 6L**, **7L**, **8A**, **9A**), which activated the c-Jun N-terminal kinase and NF-κB pathways, leading to endoplasmic reticulum stress (71). In turn, this significantly affected the insulin sensitivity of adipocytes, causing glucose dyshomeostasis in adipocytes and aberrant BG levels (**Figures 10A, B**).

In addition to the aforementioned metabolic disorders, CIH- and CSF-induced alterations of the gut microbiota, which predisposed the mice to CVDs, NDDs, and IDs (**Figures 8A**, **9A**). CIH induced periodic excitation of sympathetic and parasympathetic nerves, autonomic nervous dysfunction (72). Additionally, CIH-induced dysbiosis of the intestinal bacteria increased the homocysteine level (**Figures 6E**, **8A**), leading to endothelial damage through free oxygen radicals and nitric oxide, which caused vascular remodeling and lipid accumulation in the vascular wall (73). These changes were closely related to the development of CVD. CSF activated the HPA axis and neurons, resulting in excitatory toxicity and neuronal injury. Meanwhile, the increase abundance of Proteobacteria and the decrease abundance of Bacteroidetes due to CSF and CSF+HFD (**Figure 5D**) decreased the levels of sphingolipid (**Figures 7R**, **9A**) and brain-derived neurotrophic factor through the enterobacteria-brain axis (74), which plays a crucial role in neuron survival, differentiation, and growth. Additionally, CSF down-regulated *E. coprostanoligenes* and endogenous vitamin B (**Figures 5D**, **7S**, **9B**), resulting in decreased immunity (75) and an increased risk of IDs (**Figure 9B**).

In conclusion, CIH and CSF regulated the levels of intestinal microbes (such as *A. mucinphila, Clostridium* spp.*, Lactococcus* spp., and *Bifidobacterium* spp.) through changes in many functional metabolites, such as tryptophan, FFAs, BCAA and Bas. These can affect adipose and hepatic tissue lipid metabolism and deposition (**Table 1**). The CIH-gut microbiota-metabolite-adipose axis promoted metabolic inflammation, whereas the CIH-gut microbiota-metabolite-liver axis promoted hepatic steatosis. The CSF-gut microbiota-metabolite-adipose axis is crucial in the regulation of BAT-mediated energy metabolism.

**Table 1:** Commonalities between CIH and CSF (with or without HFD-fed) related modification on lipid metabolism through gut microbiota-metabolite-liver/adipose axis.

CIH, CSF and dietary modification on	Related mechanism of lipid metabolism disorders
Gut microbiota composition or properties	
Intestinal bacterial derivative metabolites	
Downregulation of ***Akkermansia mucinphlia***	caused intestinal mucosal damage and increased intestinal permeability (21)
Upregulation of **Firmicutes**	activated PPAR-γ to promote β-oxidation of BAT (25)
induced insufficient **BCAA**	promoted IR (71)
Upregulation of **Firmicures/Bacteroidetes**	promoted TG deposition in WAT (40)
induced insufficient **SCFAs**	inhibited protective PPAR-α to induce hepatic steatosis (52)
insufficient **BAs**	inhibited TGR5 to induce IR through decreased endogenous GLP-1 secretion (70)
Upregulation of **FFAs** due to CIH or CSF-decreased ***Clostridium* spp.** CIH-increased ***Bifidobacterium* spp.** or ***Lactobacillus* spp.**	actived pro-inflammatory TLR2 and increased expression of pro-apoptotic gene Bax in liver, WAT and BAT (39); caused hepatic steatosis due to excessive synthesis of TG and cholesterol (58); actived c-JNK and NF-κB to induced ER stress and IR (72)
Downregulation of ***Clostridium* spp.** induced insufficient **tryptophan** and **indoles**	inhibited AHR to promote IR through decreased endogenous GLP-1 secretion (32)
Downregulation of **glutamate** and **glutathione** due to CIH-increased ***Enterorhabdus* spp.** CSF-decreased ***Bacteroides* spp.**	induced excessive ROSs corelated with hepatic steatosis (50)
Upregulation of **LPS** due to CIH-increased ***Desulfovibrio* spp.** CSF-increased **Proteobacteria**	actived pro-inflammatory TLR4 and increased hepatic VLDL and lipoprotein levels (64)

BAT, brown adipose tissue; WAT, white adipose tissue; BCAA, branched-chain amino acid; SCFA, short-chain fatty acid; BA, bile acid; LPS, lipopolysaccharide; TG, triglyceride; VLDL, very low-density lipoprotein; IR, insulin resistance; PPAR, peroxisome proliferator-activated receptor; TGR5, takeda G protein-coupled receptor; GLP-1, glucagon-like peptide-1; TLR, toll-like receptor; c-JNK, c-Jun N-terminal kinase; ER, endoplasmic reticulum; AHR, aryl hydrocarbon receptor; ROS, reactive oxygen species. The red color signified up-regulation, whereas the blue color signified down-regulation.

Our study had several limitations. First, due to the interaction and synergistic regulation of multiple functional metabolites, it was difficult to identify the key metabolites regulating changes in other metabolites. Second, functional metabolites with physiological and pathological effects were not comprehensively evaluated, and many metabolites may have been missed. Third, there were a lot of speculations in the discussion correlating the metabolomic or microbiome findings with pathological and metabolic organismal changes without meticulously following in depth each finding.

Gut microbes and their downstream metabolites are important for host health, linking diet and environmental factors in that context. Regulating the changes in intestinal flora associated with CIH and CSF may constitute a new approach to the prevention and treatment of OSA-induced lipid metabolic disorders. However, this emerging area of research requires further data. Our future studies should use tools such as metabolite annotation and gene integration to correlate gene sequences with metabolomics data, to generate multi-layer datasets (mRNA transcription, protein translation, and epigenetic modification). The mechanism of regulation of lipid metabolism by the intestinal microbiota and functional metabolites should be explored in sterile animals, using engineered bacteria (metabolic enzyme knockout or overexpression) and artificial compounds (76).

## Data Availability Statement

The datasets presented in this study can be found in online repositories. The names of the repository/repositories and accession number(s) can be found below: https://www.ncbi.nlm.nih.gov/, PRJNA781373.

## Ethics Statement

The animal study was reviewed and approved by the Institutional Animal Care and Use Committee of Shanghai Jiao Tong University Affiliated Sixth People’s Hospital, China.

## Author Contributions

FW, HX, JG, and SY contributed to conception and design of the study. JJZ, FW, WH, XZ, ZW, XL, YL, and JYZ organized the database. FW, JJZ, JYZ, and HZ performed the statistical analysis. FW and JJZ wrote the first draft of the manuscript. HX, HY, FL, and JG wrote sections of the manuscript. All authors contributed to manuscript revision, read, and approved the submitted version.

## Funding

This study was supported by grants-in-aid from Shanghai Municipal Commission of Science and Technology (Grant No.18DZ2260200); InnovationProgram of Shanghai Municipal Education Commission (Grant No. 2017-01-07-00-02-E00047); National Natural Science Foundation of China (Grant Nos. 81770987, 81970870;82071030, 81770988; 82000968) and Shandong Natural Science Foundation Program (Grant No. ZR201911030252).

## Conflict of Interest

The authors declare that the research was conducted in the absence of any commercial or financial relationships that could be construed as a potential conflict of interest.

## Publisher’s Note

All claims expressed in this article are solely those of the authors and do not necessarily represent those of their affiliated organizations, or those of the publisher, the editors and the reviewers. Any product that may be evaluated in this article, or claim that may be made by its manufacturer, is not guaranteed or endorsed by the publisher.

## Supplementary Material

Supplementary Table S1 Effects of CIH on the serum negative and positive metabolic ion peaks.

Supplementary Table S2 Effects of CSF on the serum negative and positive metabolic ion peaks.

Supplementary Table S3 Effects of CIH on the serum secondary metabolites.

Supplementary Table S4 Effects of CSF on the serum secondary metabolites.

## Abbreviation

5-HT, 5-hydroxytryptamine; ATM, adipocyte macrophages; AHR, aryl hydrocarbon receptor; BA, bile acid; BG, blood glucose; BCAA, branched-chain amino acid; BAT, brown adipose tissue; CVD, cardiovascular disease; CIH, chronic intermittent hypoxia; CSF, chronic sleep fragmentation; F/B, Firmicutes/Bacteroidetes; FFA, free fatty acids; GLP-1, glucagon-like peptide-1; HFD, high-fat diet; HPLC. high-performance liquid chromatography; HPA, hypothalamic-pituitary-adrenal; ID, infectious disease; IWAT, inguinal white adipose tissue; IR, insulin resistance; IL-6, interleukin-6; KEGG, Kyoto Encyclopedia of Genes and Genomes; LPS, lipopolysaccharide; LC, liquid chromatography; log_2_FC, log_2_ fold change; MS, mass spectrometry; MCP-1, monocyte chemoattractant protein-1; MUFA, monounsaturated fatty acid; NDD, neurodegenerative disease; NS, normal sleep; NM, normoxia; OSA, obstructive sleep apnea; OTUs, operational taxonomic units; PWAT, perirenal white adipose tissue; PPAR-γ, peroxisome proliferator-activated receptor-γ; PICRUSt, Phylogenetic Investigation of Communities by Reconstruction of Unobserved States; PCR, polymerase chain reaction; PUFA, polyunsaturated fatty acid; PCA, principal component analysis; PCoA, principal coordinate analysis; QC, quality control; QIIME, Quantitative Insights into Microbial Ecology; ROS, SCFA, reactive oxygen species, short-chain fatty acid; TLR, toll-like receptor; TG, triglyceride; TNF-α, tumor necrosis factoralpha; UCP1, uncoupling protein 1; WAT, white adipose tissue.
